# Correction to “Identification of the sweet orange (*Citrus sinensis*) *bHLH* gene family and the role of *CsbHLH55* and *CsbHLH87* in regulating salt stress”

**DOI:** 10.1002/tpg2.70165

**Published:** 2025-12-17

Zi, Y., Zhang, M., Yang, X., Zhao, K., Yin, T., Wen, K., Li, X., Liu, X., & Zhang, H. (2024). Identification of the sweet orange (*Citrus sinensis*) *bHLH* gene family and the role of *CsbHLH55* and *CsbHLH87* in regulating salt stress. *The Plant Genome*, *17*, e20502. https://doi.org/10.1002/tpg2.20502

There is an error in the second sentence of “Section 2.9 Quantitative real‐time polymerase chain reaction (qRT‐PCR) analysis” where the word “kiwifruit” was used instead of “sweet orange.” The sentence “Sweet orange leaves cultured in regular media for 14 days were used as the control group (CK1, CK2, CK3), and kiwifruit leaves cultured in salt‐stressed medium for 14 days were used as the treatment group (T1, T2, T3)” has been corrected to “Sweet orange leaves cultured in regular media for 14 days were used as the control group (CK1, CK2, CK3), and sweet orange leaves cultured in salt‐stressed medium for 14 days were used as the treatment group (T1, T2, T3).”

We apologize for this error.

